# Fluorescence Correlation Spectroscopy (FCS) Unlocks the Presence of Microcystin-LR in Water

**DOI:** 10.3390/life16020264

**Published:** 2026-02-03

**Authors:** Antonio Varriale, Giovanni Ferrara, Sabato D’Auria

**Affiliations:** Istituto di Scienze dell’Alimentazione, Consiglio Nazionale delle Ricerche, 83100 Avellino, Italy; giovanniferrara1@cnr.it (G.F.); sabato.dauria@cnr.it (S.D.)

**Keywords:** *Cyanobacterial* bloom, fluorescence correlation spectroscopy (FCS), immunoassay, microcystin

## Abstract

Water is essential for human life, and access to clean water is considered a basic human right by the United Nations. Around the world, a high proportion of the population still does not have access to safe fresh water, with high impact on health. This situation perpetuates a cycle of poverty, hindering economic development and exacerbating inequality. Water is considered unsafe to drink if it is contaminated. The contamination can be categorized into three types: physical, chemical, and biological. Biological contamination arises from the presence in water of living organisms such as bacteria, viruses, algae, fungi, and parasites. Recently, the scientific community has raised the alarm on contamination caused by a large group of bacteria known as *Cyanobacteria*, which can release harmful toxins into water, including cyanotoxins like microcystin-LR (MC-LR). Currently, the standard analytical procedure for the detection and quantification of MC-LR relies on chromatography-based techniques (HPLC, LC/MS, GC/MS), immunological assays (ELISA), or protein phosphatase inhibition assays (PPIAs). In this study, we used fluorescence correlation spectroscopy (FCS) methodology to develop a competitive assay for detection of traces of the MC-LR toxin in a water solution. A conjugated form of bovine serum albumin (BSA) with MC-LR was labeled with the fluorescence dye CF488 (MC-LR BSA 488). The diffusion coefficient values of the MC-LR BSA 488 complex were investigated in the absence and in the presence of MC-LR. The change in the value of the diffusion coefficient was correlated with the concentration value of MC-LR in solution. The results showed a limit of detection (LoD) of the assay of 0.18 nM (0.18 µg/L), a value lower than the limit value (1.0 μg/L) established by the World Health Organization (WHO).

## 1. Introduction

Water is a finite resource essential to human life, agriculture and industry. Access to safe and uncontaminated water is essential for human life. However, in different parts of the world, many communities still have problems due to a lack of water, or may have access only to contaminated water, with serious resulting health risks, environmental impacts, and economic consequences [1].

Water contamination can arise from various sources, such as industrial and agricultural waste, sewage, and natural events like hurricanes and floods. Contamination is generally classified based on its origins: physical, chemical, or biological. All these types of contamination produce unsafe drinking water. Biological contamination originates from the presence in water of living organisms like bacteria, viruses, algae, fungi, and parasites. These contaminants can lead to severe health issues, including gastrointestinal illnesses, dermatological infections, and respiratory illnesses [2].

Recently, the scientific community has raised alarms about water contamination caused by a group of bacteria known as *Cyanobacteria* [3]. *Cyanobacteria* are photosynthetic prokaryotic microorganisms that live in a diverse range of environments, from freshwater to marine ecosystems. The uncontrolled growth of these bacteria due to anthropogenic factors (such as water ecosystem degradation, increased CO_2_ level in the atmosphere, etc.) led to the *Cyanobacteria* bloom phenomenon [4]. Specific genera of *Cyanobacteria* can produce and release harmful toxin molecules known as microcystins (MCs) into the water [2].

Within the MC family, the most toxic molecule is microcystin-LR (MC-LR). Due to its high toxicity, the World Health Organization (WHO) has established a provisional value for the maximum amount of MC-LR intake. It has been set for the drinking water at 1.0 μg/L and a daily intake value (TDI) of 0.04 μg/kg MC-LR/body weight [2].

Currently, the strategies used to detect and quantify MC-LR are chromatography techniques (HPLC, LC/MS, GC/MS), immunological methods (ELISA), and protein phosphatase inhibition assays (PPIAs) [4]. In addition, a methodology based on the use of Förster resonance energy transfer (FRET) to determine the presence of MCs in solution has been developed [5].

Among the fluorescence spectroscopy methodologies, fluorescence correlation spectroscopy (FCS) is a method with various applications [6,7]. Several studies have shown the effectiveness of FCS for addressing fundamental questions in biology [8,9,10,11,12,13]. Furthermore, some studies have used this technique to identify the presence of specific molecules, such as allergens, toxins, and antibiotics, in various matrices [14,15].

In this work, we present a competitive FCS assay to detect the presence of MC-LR in solution. The assay is based on th analysis of the fluctuations of fluorescence molecules present in a small observation confocal volume [6].

A conjugated form of bovine serum albumin (BSA) with MC-LR was labeled with the fluorescence dye CF488 (MC-LR BSA 488). The diffusion coefficient values of the MC-LR BSA 488 complex in both the absence and presence of MC-LR were investigated. The change in diffusion coefficient value was correlated with the concentration value of MC-LR in solution. The results showed a limit of detection (LoD) of 0.18 nM (0.18 µg/L), a value lower than the limit value (1.0 μg/L) established by the World Health Organization (WHO).

## 2. Materials and Methods

### 2.1. Materials

Microcystin-LR antibody and MC-LR were purchased from Enzo Life Sciences (Enzo Life Sciences Inc., Farmingdale, NY, USA). MC-LR BSA conjugate was purchased from Creative Diagnostics (Creative Diagnostics, Shirley, NY, USA). The fluorescent dye CF488 was supplied by Biotium (Biotium Inc., Fremont, CA, USA). The microplates (96-well), C8 lock-well maxisorp Nunc (ref. cod. 446469), were purchased from Thermo Fisher Scientific Inc. (Waltham, MA, USA). Goat polyclonal IgG-HRP secondary antibody used in the ELISA experiments was purchased from Abcam (Abcam plc., Oregon, OR, USA). Polyclonal antibody anti-Ovalbumin, used as a control in the experiments, was purchased from Covalab (Bron, France). Sephadex G25 chromatographic resin was purchased from Sigma-Aldrich (Sigma-Aldrich S.r.l., Milan, Italy). All other chemicals were commercial samples of the purest quality.

### 2.2. Preparation of the MC-LR BSA Labeling with CF488 Dye

The labeling of the MC-LR-BSA with the CF488 dye was performed according to the method described by Capo [5]. In brief, the conjugate was dissolved in 0.10 mL of 20 mM sodium phosphate buffer, pH 7.4, at a concentration of 2.0 mg/mL, and mixed with CF488 (molar dye-antibody ratio: 12:1) dissolved in dimethyl sulfoxide (DMSO). The obtained mixture was adjusted to pH 8.3, and the reaction was stirred in the dark at room temperature for one hour. After the incubation period, the labeled conjugate, MC-LR BSA 488, was purified of any unreacted CF488 dye using a Sephadex™ G25 column. The chromatography steps and the characterization of the obtained fractions were performed according to Capo [5].

### 2.3. Fluorescence Steady-State Measurements

Fluorescence steady-state experiments were performed using an ISS K2 fluorometer (ISS Inc., Champaign, IL, USA) equipped with a temperature-controlled sample holder. The stock solution of MC-LR BSA 488 was diluted to a final optical density of 0.07 O.D. at 488 nm, to prevent the inner filter effect [16]. All the measurements were performed with the excitation wavelength set at 488 nm, and emission spectra from 500 nm to 600 nm were recorded. The MC-LR BSA 488 emission spectra were recorded at 25 °C, in PBS buffer at pH 7.4, with a final volume of 750 μL.

### 2.4. Fluorescence Correlation Spectroscopy (FCS) Experiments

The FCS measurements were performed on Alba V, a dual-channel fluorescence correlation instrument from ISS (ISS Inc., Champaign, IL, USA). The instrument combines a confocal scanning microscope with FCS, and it uses avalanche photodiodes as detectors. It is equipped with a Nikon inverted microscope and a laser launcher with two lasers (488 nm and 640 nm); the output of each laser is aligned to a single-mode fiber to produce a Gaussian-profile laser beam to be delivered to the instrument. We used a Nikon high-numerical-aperture (NA) water objective (60×; NA 1.2), and focused on the sample solution. The resulting fluorescence was collected through the same objective and separated from the laser light by a dichroic mirror (Croma). A 50-μm pinhole was used in the confocal detection channel. All pinhole adjustments, shutters, optics, filter wheels, XYZ-fine positioning of the stage, and positioning of the objective were computer-controlled using the Vista Analysis Software(version 4.1) program (ISS Inc., Champaign, IL, USA). The alignment of the instrument and the characteristics of the confocal beam were validated using rhodamine 110, which has a known diffusion coefficient of 430 μm^2^/s [16,17,18]. All measurements were performed at room temperature, and the obtained data were analyzed with the Vista Analysis Software program (ISS Inc., Champaign, IL, USA).

#### 2.4.1. FCS Binding Analysis

The antibody binding measurements were performed by diluting MC-LR BSA 488 in PBS buffer at pH 7.4 to a final volume of 100 µL, then incubating with increasing concentrations of anti-MC-LR (0.0 nM–125 nM). For each concentration, before performing the FCS measurements, the samples were incubated under dark conditions and at room temperature for 30 min.

#### 2.4.2. FCS Competitive Assay

The monoclonal antibody anti-MC-LR (125 nM) was incubated for 30 min with increasing concentration of unlabeled MC-LR (from 0.1 nM to 20 nM). At the end of this incubation step, BSA-MC-LR 488 was added, incubated for 30 min at room temperature, and then FCS measurements were performed.

#### 2.4.3. Specificity of FCS Assay

To evaluate the specificity of the assay, the measurement was performed using a different antibody. For this purpose, the polyclonal antibody anti-ovalbumin (125 nM) was incubated for 30 min with 20 nM of MC-LR. At the end of this incubation step, MC-LR BSA 488 was added, and incubated for 30 min under dark conditions at room temperature. The FCS measurements were then taken.

#### 2.4.4. FCS Data Analysis

All collected data were analyzed using the Vinci Analysis Software program (ISS Inc., Champaign, IL, USA). Specifically, a fitting model based on a three-dimensional Gaussian volume and a two-species observation volume was employed (Equation (1)).

(1) $$G\left(\tau \right)=\left(\frac{2\sqrt{2}}{\pi \sqrt{\pi }\omega_{0}^{2}z_{0}\langle C\rangle }\right)\times \sum_{i=1}^{n}\frac{f_{i}}{\left(1+\frac{8D_{i}\tau }{\omega_{0}^{2}}\right)\sqrt{1+\frac{8D_{i}\tau }{z_{0}^{2}}}}exp\left[\frac{{-(V_{i}\tau )}^{3}}{\omega_{0}^{2}z_{0}\left(1+\frac{8D_{i}\tau }{\omega_{0}^{2}}\right)\sqrt{1+\frac{8D_{i}\tau }{z_{0}^{2}}}}\right]$$

In this equation, G(τ) represents the autocorrelation function, C is the concentration of the fluorescence molecule, ω_0_ is the beam waist, z_0_ is the beam height, D is the diffusion coefficient, V is the excitation volume, Π is Archimedes ‘constant, and τ is the time diffusion.

### 2.5. Data Analysis

All experiments were performed in triplicate, and results were presented in the form of mean ± standard deviation (SD). The analytical performance of the FCS assay was evaluated according to Shrivastava (LoD = 3.3 S/b) [19]. All graphics were generated using Excel 2016 Microsoft^®^ software and/or by Origin Pro^®^ 8.0 software.

## 3. Results and Discussion

MCs are cyclic heptapeptides released in water from *Cyanobacteria*. From a chemical point of view, the MC-LR, with respect to other MCs, contains arginine and leucine in the variable portion of its structure [3,5].

For our assay, we selected the monoclonal antibody anti-MC-LR, which recognizes all the variants of the four-Arg microcystins. However, we focused specifically on MC-LR due to its high prevalence in freshwater ecosystems and its significant toxicity.

**Figure 1:**
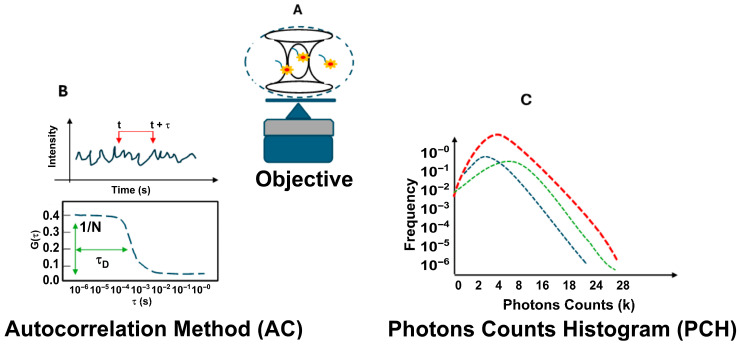
Fluorescence correlation spectroscopy (FCS) setup. In the general FCS optical setup, a laser is focused on a diffraction-limited spot using a high-numerical-aperture objective lens. Emitted light is collected through a confocal pinhole, defining a small observation volume of approximately 0.2 femtoliters (**A**). Analysis of fluctuations using autocorrelation (AC) analysis (**B**) and photon counting histogram (PCH) analysis (**C**).

Figure 1 shows the FCS optical setup used for the experiments. It includes the confocal volume size (Figure 1A) and two analysis methods: autocorrelation analysis (AC) and photon counting histogram analysis (PCH) (Figure 1B,C).

The MC-LR BSA complex was labeled with the fluorescence dye (CF488). The degree of labeling (DoL) of MC-LR BSA 488 was calculated from the absorption spectrum, according to Varriale [13]. A value of three molecules of CF488 per molecule of MC-LR BSA was calculated.

**Figure 2:**
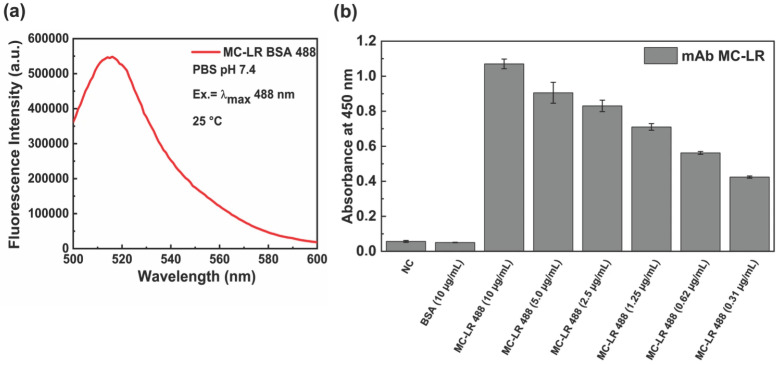
Characterization of the MC-LR-BSA-488 conjugate. Fluorescence emission spectrum obtained upon excitation at 488 nm (**a**). ELISA binding assay of monoclonal anti-MC-LR against MC-LR-BSA-488 at 25 °C. A BSA solution and a no-coating (NC) well were used as negative control (**b**).

In Figure 2a the emission spectra of the fluorescence MC-LR BSA 488 are displayed.

To evaluate the effect of the labeling process on the binding capacity of the antibody to MC-LR BSA 488, an indirect ELISA test was performed (Figure 2b). The results show that the anti-MC-LR binds the labeled conjugate MC-LR BSA 488 from 0.31 µg/mL to 10.0 µg/mL.

### 3.1. FCS Measurements: Binding Characterization

**Figure 3:**
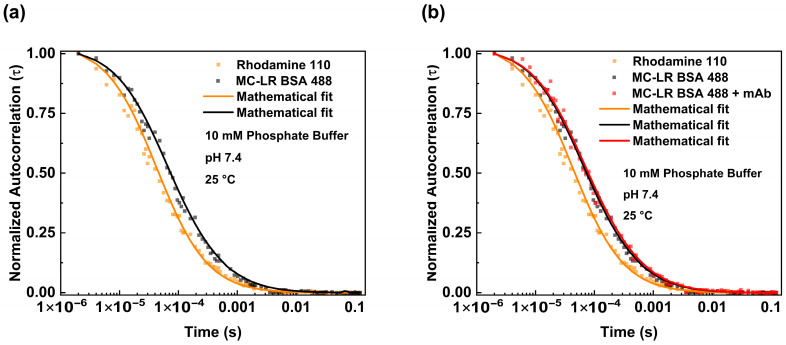
Fluorescence correlation spectroscopy measurements of MC-LR BSA 488—mAb anti-MC-LR complex. Normalized autocorrelation curve of MC-LR BSA 488 (black dots) in PBS pH 7.4 compared with the autocorrelation spectrum of Rhodamine 110 (orange dots) (**a**). Normalized autocorrelation of MC-LR BSA 488 in the absence (black dots) and presence of 125 nM of mAb anti-MC-LR (red dots), compared with the autocorrelation spectrum of Rhodamine 110 (orange dots) (**b**). The measurements were performed in PBS buffer at pH 7.4 and at 25 °C.

The binding between the antibodies and MC-LR BSA 488 was carried out in different steps. We investigated the diffusion of the MC-LR BSA 488 alone (Figure 3a black dots) and analyzed the obtained fluctuation data using the equation reported in Section 2 (3D Gaussian-2 species). We estimated a diffusion coefficient (D) value of 73.02 μm^2^/s. This value is consistent with the molecular weight of the BSA molecule, as reported in the literature, and is slightly different from the theoretical value considering the presence of MC-LR molecules [20].

Furthermore, the behavior of MC-LR/MC-LR BSA 488 in the presence of antibodies was evaluated. For this purpose, the MC-LR BSA 488 was incubated with a higher concentration of mAb anti-MC-LR. Figure 3b shows the normalized autocorrelation curves obtained from the MC-LR BSA 488 in both the absence and presence of anti-MC-LR (125 nM). The diffusion coefficient values, determined with the autocorrelation function, show a reduction in the diffusion coefficient value. Specifically, the diffusion coefficient values change from 73.02 μm^2^/s to 49.52 μm^2^/s when the antibody concentration reaches 125 nM. This change is consistent with the formation of a molecular complex similar in size to the anti-MC-LR/MC-LR BSA 488 complex (Table 1).

**Figure 4:**
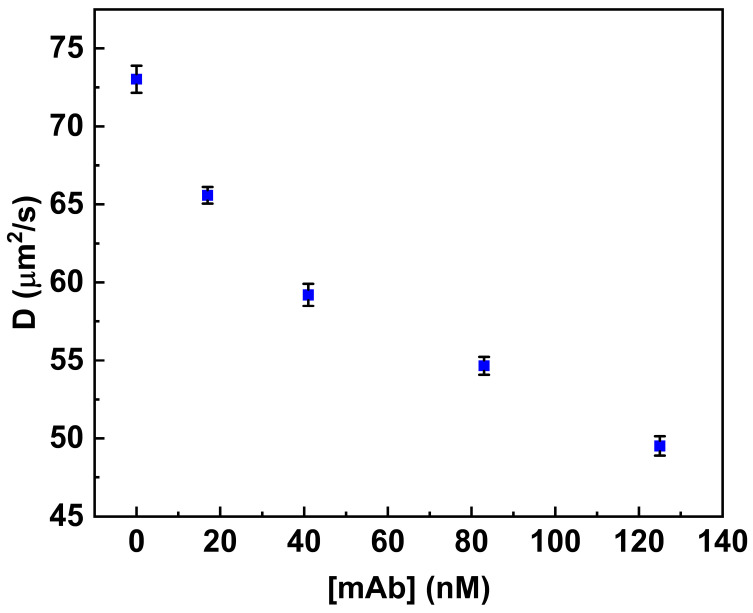
Binding of anti-MC-LR to MC-LR-BSA-488. Variation in the diffusion coefficient (D) of the fluorescent conjugate in the presence of increasing concentrations of anti-MC-LR (0.0 nM–125 nM). All measurements were performed in PBS buffer (pH 7.4) at 25 °C.

Figure 4 shows the variation in the diffusion coefficient values (see Table 1) associated with increased concentration values of anti-MC-LR in solution (from 0.0 nM to 125 nM).

### 3.2. FCS Measurements: Competitive Assay

**Figure 5:**
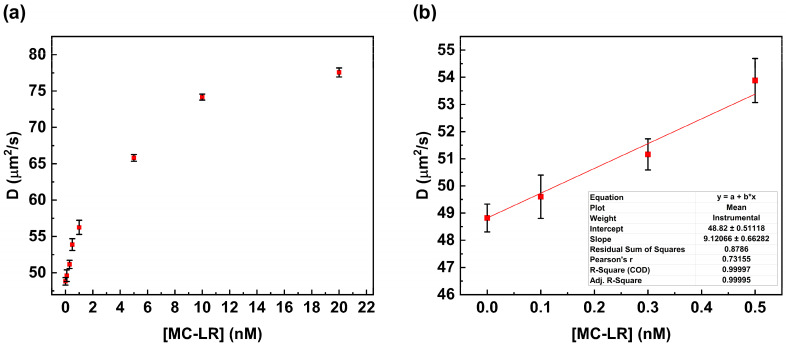
Fluorescence correlation spectroscopy competitive assay. Variation in the diffusion coefficient value in the presence of increasing concentrations of MC-LR (**a**). Calibration curve is within the range of 0.0 nM to 0.5 nM for MC-LR (**b**).

Figure 5a shows the plot of the change in the diffusion coefficient value versus MC-LR concentration. The increase in MC-LR concentration results in an increase in the value of the diffusion coefficient due to the disruption of the complex anti-MC-LR/MC-LR BSA 488. This value is close to the value calculated in the absence of the MC-LR (see Table 2).

In particular, the diffusion coefficient value changes from 48.82 µm^2^/s to 77.56 µm^2^/s. This behavior suggests complete dissociation of the anti-MC-LR/MC-LR BSA 488 complex in the presence of the MC-LR in solution.

A linear correlation was observed over the range from 0.0 nM to 0.5 nM, whereas at higher concentrations (0.5 nM to 20 nM), no linear trend was observed. The calibration curve was calculated within the linear range to determine the limit of detection (LoD) according to Shrivastava (LoD = 3.3 S/b) [19]. The LoD was calculated to be 0.18 nM (0.18 µg/L) (Figure 5b).

These results suggest that the use of high-avidity monoclonal antibodies with innovative fluorescence immunoassay allows for the detection of traces of MC-LR in solution.

## 4. Conclusions

In conclusion, the obtained results suggest the potential use of FCS as a powerful tool in the development of a new-generation analytical sensing approach to detect specific target analytes. In fact, the experimental conditions under which the FCS works can allow for the detection of very low concentrations of contaminants (nanomolar range). This requirement is becoming a central feature in the development of highly sensitive methodologies with applications in several fields (environmental, food safety, etc.). Specifically, in this study, we showed the efficacy of a competitive assaybased FCS for very sensitive detection of toxin MC-LR. The assay leverages the analysis of fluorescence fluctuations to monitor the competitive binding between free MC-LR and a labelled MC-LR-BSA 488 conjugate to a specific antibody. The amount of MC-LR detected in the solution is lower than the legal requirements imposed by the WHO.

Finally, although these results were obtained using laboratory samples, they indicate that this methodology can be suitable for practical applications. Further studies should be focused on investigating the performance of the assay in real environmental matrices, such as raw lake or river water, where the cyanobacterial bloom phenomenon occurs and high numbers of interfering substances are present. Such an experiment will be essential for validating the methodology as a reliable tool for measuring MC-LR levels in real freshwater samples.

## Figures and Tables

**Table 1:** Diffusion coefficient in the absence and in the presence of increasing concentrations of monoclonal anti-MC-LR.

[Anti-MC-LR] nM	Diffusion Coefficient µm^2^/s ± SD	Chi-Square
0.0	73.02 ± 0.87	0.83
17.0	65.58 ± 0.53	1.15
41.0	59.20 ± 0.70	0.75
125.0	49.52 ± 0.62	0.77

**Table 2:** Diffusion coefficient values of anti-MC-LR/MC-LR BSA 488 without or with an increasing concentration of MC-LR. The anti-MC-LR concentration was fixed at 125 nM.

[MC-LR] nM	Diffusion Coefficient µm^2^/s ± SD	Chi-Square
0.0	48.82 ± 0.51	0.75
0.1	49.60 ± 0.79	0.94
0.3	51.16 ± 0.57	0.74
0.5	53.88 ± 0.81	1.02
1.0	56.24 ± 0.97	0.85
5.0	65.81 ± 0.46	0.92
10.0	74.16 ± 0.41	1.12
20.0	77.56 ± 0.62	1.09

## Data Availability

The original contributions presented in this study are included in the article. Further inquiries can be directed to the corresponding author.

## Abbreviations

The following abbreviations are used in this manuscript:

FCS	Fluorescence Correlation Spectroscopy
MC-LR	Microcystin-LR
BSA	Bovine Serum Albumin
WHO	World Health Organization
AC	Autocorrelation analysis
PCH	Photon Counting Histogram Analysis
OVA	Ovalbumin
ELISA	Enzyme-Linked Immunosorbent Assay
